# Untreated caries and serum vitamin D levels in children and youth of the United States: NHANES 2013-2014

**DOI:** 10.1590/0103-6440202305123

**Published:** 2023-03-06

**Authors:** Lilian Rigo, Augusto Bacelo Bidinotto, Fernando Neves Hugo, Matheus Neves, Juliana Balbinot Hilgert

**Affiliations:** 1 Graduate Program in Dentistry, ATITUS, Passo Fundo, Brazil; 2 Department of Preventive and Social Dentistry, Federal University of Rio Grande do Sul, Porto Alegre, Brazil

**Keywords:** 25-hydroxyvitamin D, 25(OH)D, vitamin D, dental caries, National Health and Nutrition Examination Survey

## Abstract

This study aimed to investigate the association between serum vitamin D levels and untreated caries and determining factors in children and youth. Methodology: This cross-sectional study used data from the National Health and Nutrition Examination Survey (2013-2014). In total, 3,072 participants with ages ranging from 1 to 19 years were included in our sample. The main dependent variable, untreated caries, was defined as having at least one untreated carious surface in any tooth. Serum concentration of 25-hydroxyvitamin D [25(OH)D] was categorized into four groups: ≥75 nmol/ml, 50-74.9 nmol/ml, 25-49.9 nmol/ml, and <25 nmol/ml. Data were analyzed using a binary logistic regression. Results: For children aged 1-5 years, age (OR = 1.68, 95% confidence intervals (95% CI) 1.38-2.04) and low levels of vitamin D (25-49.9 nmol/ml, and <25 nmol/ml: OR = 2.55, 95% CI 1.06-6.13) were associated with untreated caries. For children aged 6-11 years, low levels of vitamin D (50-74.9 nmol/ml: OR=1.45, 95% CI 1.16-1.82) remained associated with untreated caries. No associations were found in those between 12 and 19 years of age. Conclusion: Our findings show an association between low levels of 25(OH)D and untreated caries in children between 1 and 11 years of age, suggesting that this nutrient might interfere in the caries process.

## Introduction

Dental caries remains highly prevalent and a worldwide public health problem that affects children, youth and adults ^1^ ^-^ ^3^. There are approximately 2.3 billion people presenting untreated caries in permanent teeth and 532 million in primary teeth ^1^. Caries has well-known determinants, such as sucrose ^4^ ^-^ ^6^ and inadequate oral hygiene ^7^. Another possible determinant is Vitamin D, which appears to reduce the risk of caries ^8^. Vitamin D, together with parathyroid hormone and calcitonin, is one of the most important biological regulators of calcium metabolism ^8^. Its deficiency, defined as 25-hydroxyvitamin D [25(OH)D]<30 nmol/L is a common risk factor for numerous diseases associated with growth hormones ^8^ ^).^ In addition to this potential mechanism of action on caries, vitamin D is also related to the production of antimicrobials, cathelicidins and defensins (antimicrobial peptides [AMPs]) in salivary glands and ductal cells, which have broad antimicrobial activity ^9^ ^,^ ^10^ ^).^ Thus, vitamin D could reduce the risk of caries by means of these peptides ^10^ ^-^ ^12^.

Vitamin D deficiency has been reported to be associated with tooth decay ^9^ ^,^ ^13^ ^-^ ^18^. However, it should be pointed out that many of these studies were carried out with non-representative samples. A systematic review of randomized controlled trials highlighted that exposure to vitamin D early in life can play an important role in the prevention of caries ^13^. Data from US adolescents ^18^ and adults ^17^ show that vitamin D deficiency was associated with higher prevalence of caries.

Therefore, this study aims to investigate the association between untreated caries and serum vitamin D levels and determining factors in children and youth, using the measurements of serum 25(OH)D levels from the data of the study conducted by NHANES from 2013-2014 in the United States.

## Methodology

### Study population and design

The National Health and Nutrition Examination Survey (NHANES) is a continuous nationally representative cross-sectional study, carried out in biennial cycles, which aims to assess the health and nutrition of the non-institutionalized civilian population in the United States using stratified and multistage probabilistic cluster sampling with four stages. The primary sampling unit (PSU) is selected in the first stage, in which a county is randomly selected from the list of all counties in the US (in some cases, adjacent counties are combined to keep PSU population above a minimum size). In the next step of sampling, a census block, or a group of census blocks are randomly selected. In the third stage, dwelling units inside these census blocks are randomly selected. Finally, in the last stage, an individual within a selected dwelling united is selected, respecting subsampling specifications based on sex, age, race and Hispanic origin, and income.

Participants were interviewed in their homes to obtain information on their health history, health behaviors and risk factors. They were then referred to a mobile examination center (MEC), where clinical examinations, nutritional interviews and the collection of biological specimens were performed ^19^. Informed consent was obtained from the participants, and the National Center for Health Statistics Research Ethics Committee approved the protocol.

In this study, we used data from cycle 2013-2014 of NHANES. Participants aged 1-19 years who received an oral examination and in whom serum 25(OH)D blood tests were carried out were included in our study ^20)^ ^.^ Of a total sample of 9,813 participants who were interviewed, a subsample of 3,072 participants was eligible for this study, which included 776 individuals aged 1-5 years, 1,054 aged 6-11 years and 1,242 aged 12-19 years.

### Procedures for data collection - Measurements of variables

### Measurement of untreated caries - outcome variable

Oral examinations were carried in the MEC, in a room fitted with dental lights, a dental chair and compressed air, with the aid of dental mirrors and explorers. The examiners were licensed dentists, which were previously trained in the NHANES examination protocol. Caries was diagnosed using visual and tactile criteria to assess the presence of lesions in each dental surface. Examiner agreement ranged between 0.82-0.90 ^21^ ^).^ Participants were categorized as having untreated caries if they presented at least one carious surface (i.e. codes 0-4 in surface condition component of the oral examination). This definition of code pertains specifically to the NHANES Oral Health Examination documentation. These numbers indicate lesion location, not severity. In any given tooth, code 0 mean lingual surface caries, code 1 means occlusal/incisal caries, code 2 means facial surface caries, code 3 means mesial caries and code 4 means distal caries. Teeth are marked with any combination of these numbers (e.g., a tooth with mesial/oral/distal lesion will be recorded as 134). These codes do not indicate lesion severity, even though NHANES diagnostic criteria divides caries lesions between “frank lesions”, which are easily detected as gross cavitation, and incipient lesions, which can be subdivided into three categories according to location, each with the following special diagnostic considerations:

1) Pits and fissures on occlusal, facial and lingual surfaces:

These areas are classified as carious when the explorer catches after insertion with moderate, firm pressure, accompanied by either a softness at the base of the area and/or an opacity adjacent to the area providing evidence of undermining or demineralization. In other words, a deep pit or fissure in which the explorer catches is not sufficient evidence of decay without one or both of the following:

i) Softness at the base of the area, and

ii) Opacity adjacent to the area providing evidence of undermining or demineralization.

2) Smooth areas on facial (labial or buccal) or lingual surfaces

These areas are carious if they are decalcified or if there is a white spot as evidence of subsurface demineralization and if the area is found to be soft by:

i) Penetration with the explorer, or

ii) Scraping the area with the explorer.

Visual evidence of demineralization is not enough to diagnose caries.

3) Proximal surfaces

i) When areas are accessible to direct visual and tactile examination, i.e., when there is no adjacent tooth, the same criteria as that used for smooth areas on facial or lingual surfaces are used.

ii) When areas are not available to direct examination, other criteria must be applied.

a) On anterior teeth, trans-illumination can serve as a useful aid in discovering proximal lesions. Trans-illumination is achieved by placing a mirror lingually and positioning the examining light so that it passes through the teeth and reflects into the mirror. If a characteristic shadow or loss of translucency is seen on the proximal surface, then this is indicative of caries on the surface. Ideally, the actual diagnosis should be confirmed by detecting a break in the enamel surface with the explorer; however, clear visualization of a lesion by transillumination can justify a positive diagnosis.

b) On posterior teeth, however, visual evidence alone, such as undermining under a marginal ridge, is not sufficient proof for diagnosing a proximal lesion. A positive diagnosis is made only if a break in the enamel surface can be detected with the explorer.

In this study, we used the prevalence of untreated caries, which represents the current prevalence of the disease. While many oral epidemiology studies use dmft/DMFT scores (or dmft/DMFT>0), it is important to highlight that it represents the lifetime prevalence of caries and does not reflect the burden of oral disease because it incorporates both treated and untreated caries. We chose to use prevalence of untreated caries, in an attempt to assess the current status of the participants in relation to dental status, and not their history of disease.

### Laboratory measures for blood collection - serum vitamin D levels

Serum 25(OH)D levels were measured using blood samples collected from participants at the MEC. The serum concentration of 25(OH)D was determined and analyzed at the National Center for Environmental Health, Center for Disease Control and Prevention, using a DiaSorin radioimmunoassay (Stillwater, MN). Serum levels of 25(OH)D (25OHD2 + 25OHD3, nmol/L) were classified into four categories: <25 nmol/mL, 25-49.9 nmol/mL, 50-74.9 nmol/mL and ≥75 nmol/mL ^17^ ^,^ ^22^.

### Covariate measurements

The covariates included in our analysis were: sex (male/female); age (in years); race/ethnicity (Mexican-American, Hispanic, non-Hispanic white, non-Hispanic black, non-Hispanic Asian and other races, including multiple races); Family income/poverty line ratio (PIR) (less than 1, above 1); frequency of tooth brushing (up to once a day, 2 or more times a day).

### Theoretical-conceptual model

**Figure 1:**
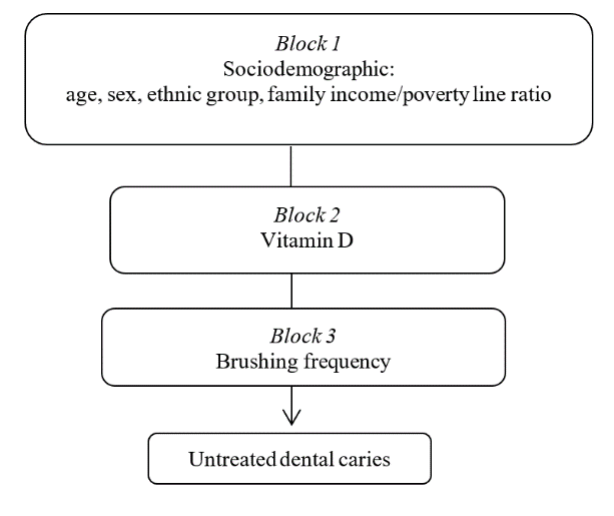
Theoretical model for untreated caries in children and youth, NHANES-2013-2014.

A theoretical-conceptual model with three blocks was used to guide the selection of exposures potentially associated with untreated caries (Figure 1). Block 1 included sociodemographic characteristics: sex, age in years, ethnic group and family PIR. Block 2 consisted of serum vitamin D levels. Block 3 consisted of oral health behaviors, including the daily frequency of tooth brushing.

### Statistical analysis of the data

All analyses were performed with MEC examination sample weights, in accordance with NHANES analytic guidelines. ^(^ ^26^ Descriptive statistics were summarized by absolute and relative frequencies. The differences between groups were assessed using the chi-square test. Odds ratios for the presence of untreated caries were estimated using logistic regression models and are presented alongside their 95% Confidence Intervals. Analysis was performed using a hierarchical approach based on the conceptual model shown in Figure 1. This approach consisted of the use of univariate logistic regression to estimate the relationship between each covariate and the outcome. Multivariable logistic regression models were then performed sequentially for each block of the conceptual model. Variables presenting p<0.20 were retained into subsequent blocks and the in the final, fully adjusted model. Analyses were performed using R 4.0.3 (R Core Team, Vienna) and the package survey 4.0.

## Results

The prevalence of untreated caries among 776 children aged 1-5 years was 11.1%. Among the 1,054 children aged 6-11 years, the prevalence of untreated caries was 17.3%. Finally, in the 1,242-youth aged 12-19 years, the prevalence of untreated caries was 21.7%. The results of the logistic regressions, according to the age groups 1-5 years, 6-11 years and 12-19 years are presented in Tables 1, 2 and 3, respectively.

In children aged 1-5 years, age (OR = 1.68, 95% CI 1.38-2.04) and low levels of vitamin D (<25 nmol/mL, 25-49.9 nmol/mL) (OR = 2.55, 95% CI 1.06-6.13) were associated with the prevalent untreated caries (Table 1). In this group, only 3 children (0.2%) were part of the 'severely deficient' category, thus, it was decided to combine this category with the 'deficit' category (n=67, 6.3%). In children aged 6-11 years, vitamin D and race/ethnicity were also associated with prevalent untreated caries. Vitamin D levels between 25-49.9 nmol/mL (OR = 1.45, 95% CI 1.16-1.82); other races, multiracial (OR=0.25, 95%CI: 0.1-0.64) were associated with untreated caries. In participants aged 12-19 years greater PIR (OR = 0.79, 95% CI 0.69-0.90) and Asian non-Hispanic ethnic group (OR = 0.54, 95% CI 0.35-0.84) were independently associated with untreated caries (Table 3), while vitamin D levels were not.

**Table 1:** Crude and adjusted odds ratio (OR) for untreated dental caries (primary teeth) in children aged 1-5 years and 95% confidence intervals (95% CI), NHANES 2013-2014, 2020 (n = 776).

	N (%)	Chi-square	p-value*	Crude	Adjusted OR (95%CI)
				OR (95%CI)	
BLOCK 1					
Sex					
Male	402(48.8)	41(10.5)	0.620	1 1.13(0.71-1.79	-
Female	374(51.2)	52(11.7)			
Age					
1	158(18.7)	0(0.0)	<0.001	1.69 (1.40-2.03)	1.68 (1.38-2.04)
2	198(21.9)	15(5.9)			
3	137(20.9)	23(15.1)			
4	139(18.9)	27(15.9)			
5	144(19.5)	28(18.9)			
*Ethnic group*					
Mexican american	189(20.0)	31(17.9)		1	
Other hispanic	92(9.6)	9(9.5)		0.48(0.20-1.15	0.50(0.20-1.22)
Non-hispanic white	204(46.7)	15(7.8)		0.39(0.16-0.95	0.46(0.17-1.25)
Non-hispanic black	172(12.9)	25(13.6)		0.72(0.37-1.39)	0.63(0.30-1.31)
Non-hispanic asian	52(3.8)	6(11.1)		0.57(0.13-2.54	0.49(0.11-2.18)
Other race- including multi-racial	67(6.9)	7(11.6)		0.60(0.31-1.16)	0.53(0.25-1.13)
PIR familiar					
≤ 1	302(34.6)	41(12.8)	0.462	1	-
> 1	423(65.4)	45(10.2)		0.85(0.66-1.11)	
					
BLOCK 2 -					
Vitamin D					
≥75 nmol/mL	335(47.6)	30(9.4)	0.021	1	1
50-74.9 nmol/mL	371(45.9)	47(11.1)		1.20(0.71-2.00)	0.97(0.55-1.70)
<49.9 nmol/mL	70(6.5)	16(22.9)		3.10(1.37-6.99)	2.55(1.06-6.13)
BLOCK 3 -					
Frequency of toothbrushing		-			
Once per day or less	127(32.8)				
Twice a day or more	282(67.2)				

* Chi-square test;

OR - Chance Ratio; 95% CI - 95% confidence interval. Adjusted for variables: sex, age, ethnic, PIR familiar and vitamin D.

**Table 2:** Crude and adjusted odds ratio (OR) for untreated dental caries (primary and permanent teeth) in children and youth aged 6-11 years and 95% confidence intervals (95% CI), NHANES 2013-2014, 2020 (n = 1.054).

	N (%)	Chi-square	p-value*	Crude	Adjusted OR (95%CI)
				OR (95%CI)	
BLOCK 1					
Sex					
Male	551(52.9)	99(17.2)	0.946	1 1.01(0.66-1.55)	-
Female	503(48.1)	95(17.4)			
Age					
6	184(16.2)	30(13.5)	0.514	1 1.03(0.93-1.13)	-
7	172 (15.8)	43 (20.7)			
8	160(16.5)	30(16.1)			
9	166(15.2)	32(17.4)			
10	185(18.2)	35(19.7)			
11	187(18.1)	24(16.3)			
*Ethnic group*					
Mexican american	251(18.6)	52(20.9)	0.186	1	1
Other hispanic	100(7.2)	16(16.0)		0.72(0.41-1.27)	0.75(0.43-1.31)
Non-hispanic white	284(51.4)	50(16.4		0.74(0.41-1.35	0.80(0.44-1.46)
Non-hispanic black	275(13.4	54(20.2)		0.96(0.58-1.57)	0.97(0.57-1.63)
Non-hispanic asian	75(4.3)	16(19.8)		0.94(0.43-2.05	0.94 (0.43-2.07)
Other race- including multi-racial	69(5.1)	6(6.0)		0.24(0.10-0.60)	0.25(0.10-0.64)
PIR familiar					
≤ 1	382(29.3)	85(22.5)	0.069	1	-
> 1	614(70.7)	102(15.8)		0.86(0.70-1.06)	
BLOCK 2					
*Vitamin D*					
≥75 nmol/mL	248(31.6)	38(13.6)	0.042	1	1
50-74.9 nmol/mL	591(54.0)	113(19.3)		1.53(1.22-1.91)	1.45(1.16-1.82)
25-49.9 nmol/mL	200(13.6)	39(17.5)		1.35(1.02-1.79)	1.21(0.90-1.64)
<25 nmol/mL	15(0.8)	4(23.6)		1.97(0.41-1.07)	1.79(0.29-11.03)
BLOCK 3					
Frequency of toothbrushing					
Once per day orless	336(35.2)	77(20.3)	0.121	1	-
Twice a day or more	681(64.8)	105(15.3)		0.71(0.47-1.07)	

* Chi-square test

OR - Chance Ratio; 95% CI - 95% confidence interval. Adjusted for variables: sex, age, ethnic, PIR, frequency of toothbrushing and vitamin D.

**Table 3:** Crude and adjusted odds ratio (OR) for untreated dental caries (primary and permanent teeth) in youth aged 12-19 years and 95% confidence intervals (95% CI), NHANES 2013-2014, 2020 (n = 1.242).

	N (%)	Chi-square	Value-p*	Crude	Adjusted
				OR (IC95%)	OR (IC95%)
BLOCK 1					
Sex					
Male	624(51.5)	147(23.4)	0.217	1 0.81(0.59-1.12)	-
Female	618(48.5)	116(19.8)			
Age					
12	163(13.2)	30(18.5)	0.340	1 1.11(1.01-1.22)	1 1.11(1.01-1.20)
13	142 (10.3)	24(14.7)			
14	174(13.9)	35(19.3)			
15	155(13.3)	30(19.6)			
16	177(13.3)	38(22.3)			
17	132(10.5)	30(20.4)			
18	168(15.0)	39(27.5)			
19	131(10.9)	37(29.9)			
*Ethnic group*					
Mexican american	303(15.7)	70(24.9)	0.217	1	1
Other hispanic	132(6.9)	22(14.9)		0.53(0.27-1.05)	0.52(0.24-1.13)
Non-hispanic white	324(54.9)	70(22.3)		0.87(0.51-1.46)	1.24(0.76-2.01)
Non-hispanic black	286(13.1)	70(24.7)		0.99(0.58-1.67)	0.96(0.54-1.70)
Non-hispanic asian	126(4.5)	18(13.8)		0.48(0.31-0.75)	0.54 (0.35-0.84)
Other race- including multi-racial	71(4.9)	13(12.5)		0.43(0.20-0.93)	0.56(0.28-1.13)
PIR familiar					
≤ 1	416(25.1)	313(72.8)	0.162	1	1
> 1	734(74.9)	138(19.3)		0.80(0.71-0.91)	0.79(0.69-0.90)
BLOCK 2					
*Vitamin D*					
≥75 nmol/mL	179(22.6)	29(15.7)	0.159	1	1
50-74.9 nmol/mL	596(50.0)	126(23.4)		1.64(0.94-2.84)	1.63(0.87-3.06)
25-49.9 nmol/mL	402(23.9)	91(23.0)		1.61(0.94-2.76)	1.55(0.81-2.97)
<25 nmol/mL	65(3.0)	17(27.2)		1.61(0.94-2.76)	1.74(0.75-4.04)
BLOCK 3					
Frequency of toothbrushing					
Once per day or less	402(34.2)	73(18.1)	0.284	1	-
Twice a day or more	916(65.8)	177(22.3)		1.30(0.82-2.07)	

* Chi-square test

OR - Chance Ratio; 95% CI - 95% confidence interval. Adjusted for variables: sex, age, ethnic, PIR, frequency of toothbrushing and vitamin D.

## Discussion

Our results showed an association between untreated caries and low vitamin D levels in children aged 1 to 11 years from the NHANES. Age was a determinant of caries in young children, while the presence of deciduous teeth and lower PIR were associated with untreated caries in youth. We certainly did not select our outcome based on the existence (or not) of significant associations, as this is a hypothesis-driven study, and we would report findings irrespective of the existence of significant associations. In addition, our decision to use untreated caries is due to the cross-sectional design. Using dmf/DMFT means incorporating measurement bias, since it incorporates past disease experience, as there is no information about when each restoration was placed. Thus, we opted to analyze untreated caries as it reflects current disease experience, which certainly is much more likely to be related with current Vitamin D levels in the case this association is existent.

An association between low serum levels of vitamin D and untreated caries was found in all age strata in the unadjusted models. However, after adjustment, low vitamin D remained associated with untreated caries only in children aged 1-5 and 6-11 years. Children aged 1-5 years with the lowest levels of serum vitamin D were 2.55 times more likely to have untreated caries than children with serum levels ≥75 nmol/mL. There was no attempt to torture data in order to have results that fitted to our hypothesis. Indeed, this was done because of data distribution. There were only 3 children aged 1 to 5 (0.2%) in the 'severely deficient' category. This means that test assumptions were not met and that we had to combine this category with the 'deficit' category, which resulted in a category with 67 children, or 6.3%, meaning that now test assumptions were met. Thus, the results need to be interpreted accounting for the fact that, in children aged 1 to 5, 'severely deficient' and 'deficient' categories were collapsed, as there were only 3 children presenting severe vitamin D deficiency. This means that test assumptions were not met and that categories had to be collapsed in order to allow statistical analysis. Whereas children aged 6-11 years in the group with serum levels of 25(OH)D between 50-74.9 nmol/mL were 1.45 times more likely to be affected by untreated caries. These findings are consistent with those of other studies carried out in children ^13^ ^,^ ^24^. Similar results, in terms of magnitude, were found in studies carried out in Canada and Sweden, in which children with higher levels of 25(OH)D were less likely to have dental caries ^24^ ^,^ ^25^.

Our results also corroborate the findings of a study that evaluated serum levels of 25(OH)D and its association with the occurrence of dental caries among North American adults from the NHANES 2007-2008 ^17^. Taken together, the findings of these studies are supportive of a relevant association between low levels of vitamin D and the prevalence of caries in children and adults. The mechanisms by which insufficiency of vitamin D interfere in the caries process remains uncertain, though. The purported mechanisms of vitamin D on caries might be related to its important effect as a regulator of calcium metabolism ^8^. Vitamin D is also plays a role in innate salivary immunity. In specific, vitamin D is key in the production of antimicrobial peptides (cathelicidins and defensins) in salivary glands and ductal cells and have broad antimicrobial activity ^9^ ^,^ ^10^. In turn, these peptides have a potential in reducing the caries-associated virulence of oral biofilms ^10^ ^-^ ^12^.

Results need to be interpreted accounting for potential sampling bias, since some groups might be overrepresented in the sample. The NHANES protocol incorporates measures to reduce this source of bias, including multiple attempts to contact potential participants and provides researchers with sampling weights that were used in the analysis of this study. Another limitation is related with the seasonality in 25(OH)D levels. Additional limitations include the lack of adjustment for important confounders such as milk intake (milk is fortified in the US), use of fluorides, and sugar intake, which might have led to misleading findings. An additional limitation is that no proxy for access to care/dental access was included as a covariate. Finally, the findings of this study are to be taken with caution because of its cross-sectional design, which precludes the determination of cause-and-effect associations. In addition, it was not possible to verify the influence of other variables, such as exposure to sunlight, on the relationship between serum 25(OH)D levels and dental caries. It was also not possible to verify the children's exposure to different fluoride sources. Therefore, the results are valid, but must be viewed with reservations due to the limitations mentioned.

## Conclusion

The findings presented in this research report suggest an important association between 25(OH)D insufficiency and untreated caries in children between 1 and 11 years of age, suggesting that this important nutrient might interfere in the caries process. Also, the results suggest that vitamin D is more associated with caries in children and youth with primary teeth, and future research to elucidate the underlying mechanisms are needed. Research is needed to identify if 25 (OH) D has the potential to be used as a caries prevention agent and to is associated with better oral development and health throughout life.
